# Supplementary material for: Controlling Selenization Equilibrium Enables High-Quality Kesterite Absorbers for Efficient Solar Cells
Source: Nat Commun. 2023 Oct 20;14:6650. doi: 10.1038/s41467-023-42460-7 (PMC10589234; doi:10.1038/s41467-023-42460-7)
Supplement: Supplementary file 1 — Supplementary Information [file 41467_2023_42460_MOESM1_ESM.pdf]

Supplementary information

Controlling selenization equilibrium enables high-quality Kesterite absorbers for efficient solar cells

Xiao Xu[1,2], Jiazheng Zhou[1,2], Kang Yin[1,2], Jinlin Wang[1,2] Licheng Lou[1,2], Menghan Jiao[1,2], Bowen Zhang[1,2], Dongmei Li[1,2,3], Jiangjian Shi[1*], Huijue Wu[1], Yanhong Luo[1,2,3*] & Qingbo Meng[1,3,4*]

[1]Beijing National Laboratory for Condensed Matter Physics, Institute of Physics, Chinese Academy of Sciences (CAS), Beijing 100190, P. R. China

[2]School of Physical Sciences, University of Chinese Academy of Sciences, Beijing 100049, P. R. China

[3]Songshan Lake Materials Laboratory, Dongguan, Guangdong 523808, P. R. China

[4]Center of Materials Science and Optoelectronics Engineering, University of Chinese Academy of Sciences, Beijing 100049, P. R. China

These authors contributed equally: Xiao Xu, Jiazheng Zhou

[*]Corresponding authors: shijj@iphy.ac.cn; yhluo@iphy.ac.cn; qbmeng@iphy.ac.cn

Supplementary Notes ········ Page 4

- Supplementary Note 1: Simulation details

Supplementary Figures ········ Page 6

- Supplementary Figure 1: Geometric construction and Se concentration evolutions in single-zone system (a, c, scale: mm) and dual-zone system (b, d, scale: cm)
- Supplementary Figure 2: Frontal and side-view of the dual-temperature zone selenization system used in this work
- Supplementary Figure 3: Time-dependent temperature evolution of Se source (full line) and the precursor film (dash line)
- Supplementary Figure 4: Optical microscope images of SG, Partial SLSG, SLSG samples
- Supplementary Figure 5: SEM EDX characterizations of SG-540℃-200s and SLSG-540℃-200s samples
- Supplementary Figure 6: Photographs of precursor films before selenization and the selenized samples (SG and SLSG) in-situ taken during the selenization process (540 °C for 300 s)
- Supplementary Figure 7: XRD patterns of SG and SLSG samples in different intermediate stages
- Supplementary Figure 8: Raman spectra of (a) Partial SLSG-400 ℃ and (b) Partial SLSG-500 ℃
- Supplementary Figure 9: XPS spectra of SG and SLSG
- Supplementary Figure 10: The evolution of morphology of ACZTSSe films in SG and SLSG routes
- Supplementary Figure 11: The schematic of liquid Se assisted selenization of ACZTSSe
- Supplementary Figure 12: Time-dependent temperature evolution designed in volatilization optimization experiments of SLSG selenization
- Supplementary Figure 13: Optimization of SLSG-O selenization
- Supplementary Figure 14: Raman spectrum of the bottom amorphous layer in SLSG

sample

- Supplementary Figure 15: Voltage-dependent photovoltage lifetimes of SG and SLSG-O samples
- Supplementary Figure 16: DLCP and C-V results of SLSG and SLSG-O samples
- Supplementary Figure 17: Temperature-dependent PL spectra of SG and SLSG-O samples
- Supplementary Figure 18: Certification report of the champion ACZTSSe solar cell given by NPVM
- Supplementary Figure 19: Cross-sectional SEM images of the completed cells with the Kesterite absorber fabricated by different methods
- Supplementary Figure 20: External quantum efficiency (EQE) spectrum of the cell
- Supplementary Figure 21: Certification report of the large-area solar cell given by NPVM

Supplementary Tables ·· Page 27

- Supplementary Table 1: Detailed parameters used in the simulation
- Supplementary Table 2: The detailed performance data of SG, SLSG, and SLSG-O cells

Supplementary References··· Page 30

Supplementary Note 1: Simulation details

Se vapor distribution in single-zone graphite box system and dual-temperature zone selenization system were simulated using COMSOL. The detailed simulation parameters of these two systems are listed in Table S1 and the results are shown in Supplementary Figure 1.

In case of single-zone graphite box system, The Se droplet is placed in a closed space (graphite box), and the initial temperature of the simulation was set to be 550°C. The simulation does not involve the heating-up process and carrier gas. Under this circumstance, the transport of Se vapor is dominated by the diffusion behavior. The shrinkage of Se droplet has been considered here. The geometric configuration of the simulation is given in Supplementary Figure 1(a). In case of dual-temperature zone system, Se source is a boat filled with elemental Se and assumed to own constant volatilization surface area. The distance between Se source and precursor is fixed to be 210 mm. The initial temperature of this simulation was also set to be 550°C and a constant flux of carrier gas is added to transport Se vapor from Se source to precursor. And for simplification, two-dimensional (2D) frontal section was used in this simulation (Supplementary Figure 1(b)).

The initial partial pressure of liquid surface is fixed to be the saturation partial pressure of Se at 550 °C. And the saturation partial pressure of Se is determined by the Antoine equation as in Supplementary Equation (1),[1] where p is the vapor pressure, T is temperature and A, B and C are component-specific constants.

$$\log_{10} p = A - \frac{B}{C+T} \quad (1)$$

The diffusion coefficient D is determined by Supplementary Equation (2),[2] where M_A and M_B are ordinary mol. weights of two gases (N_2 and Se_x), P is total pressure (1 atm), V_A and V_B are molal volumes at the normal boiling point.

$$D = \frac{0.0043T^{3/2}\sqrt{\frac{1}{M_A}+\frac{1}{M_B}}}{(V_A^{1/3}+V_B^{1/3})P} \quad (2)$$

The distribution of airflow field is simulated by Laminar Flow application mode basing on Supplementary Equation (3).[3] **u** is flow field, **I** is unit matrix, **F** is volume force, ρ is density

of the fluid, P is the atmosphere pressure, μ is dynamic viscosity of the fluid.

$$\begin{cases} \rho \frac{\partial \mathbf{u}}{\partial t} + \rho(\mathbf{u} \cdot \nabla)\mathbf{u} = \nabla \cdot [-P\mathbf{I} + \mathbf{K}] + \mathbf{F} \\ \rho \nabla \cdot \mathbf{u} = 0 \\ \mathbf{K} = \mu(\nabla \mathbf{u} + (\nabla \mathbf{u})^{\mathrm{T}}) \end{cases} \tag{3}$$

The transport of Se vapor is simulated by Transport of Diluted Species application mode basing on Supplementary Equation (4).[4] c_i is s the concentration of the species, $\mathbf{J}_\mathrm{i}$ is the mass flux relative to the mass averaged velocity, $\mathbf{u}$ is flow field, R_i is transport of diluted species, and D_i is the diffusion coefficient.

$$\begin{cases} \frac{\partial c_i}{\partial t} + \nabla \cdot \mathbf{J}_i + \mathbf{u} \cdot \nabla c_i = R_i \\ \mathbf{J}_i = -D_i \nabla c_i \end{cases} \tag{4}$$

The temperature field is simulated by Heat Transfer in Solids and Fluids application mode basing on Supplementary Equation (5).[5] ρ is the density, C_p is the specific heat capacity at constant pressure, T is the absolute temperature, $\mathbf{u}$ is the velocity vector, $\mathbf{q}$ is the heat flux by conduction, k is the thermal conductivity, Q is the heat sources.

$$\begin{cases} \rho Cp \frac{\partial T}{\partial t} + \rho Cp \mathbf{u} \cdot \nabla T + \nabla \cdot \mathbf{q} = Q + Q \\ \mathbf{q} = -k \nabla T \end{cases} \tag{5}$$

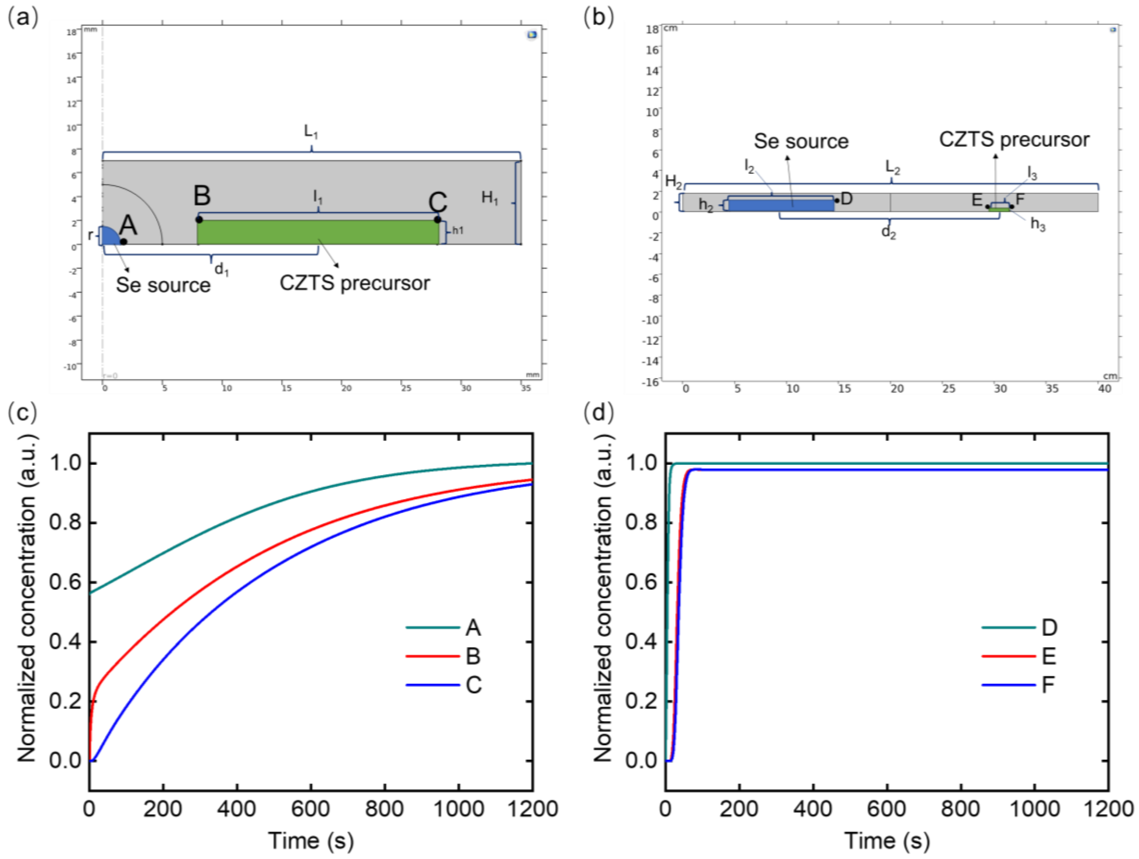

Supplementary Figure 1. Geometric construction and Se concentration evolutions in single-zone system (a, c, scale: mm) and dual-zone system (b, d, scale: cm). Site A, B, C, D, E and F are the sampled positions in the simulation. The results indicate that the dual-temperature zone system can provide much more uniform Se distribution than that of single-zone system.

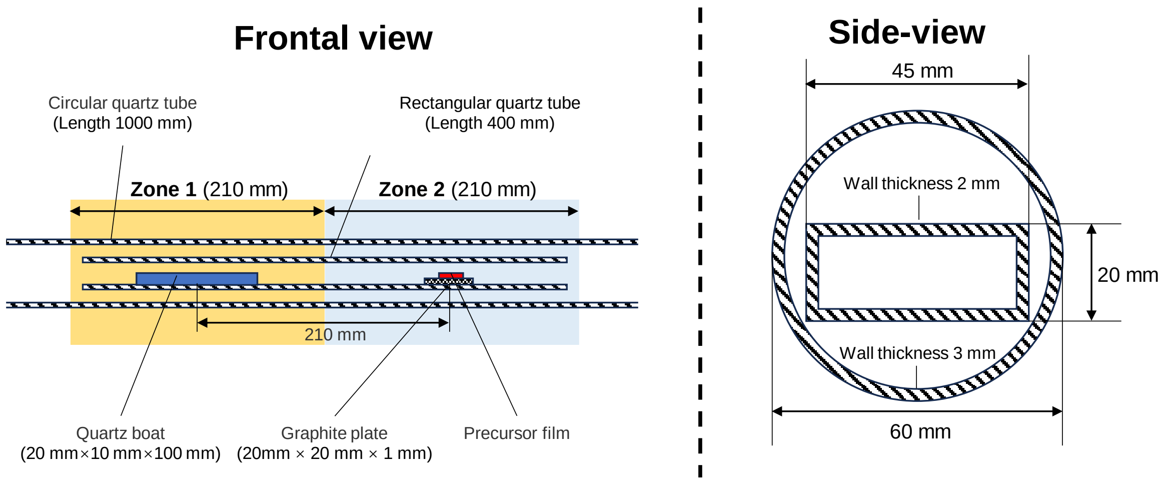

Supplementary Figure 2. Frontal and side-view of the dual-temperature zone selenization system used in this work. A rectangle quartz tube is placed into circular quartz tube to act as the reaction chamber with smaller space and higher operation convenience. Precursor films are placed on a thin graphite plate for more uniform heating.

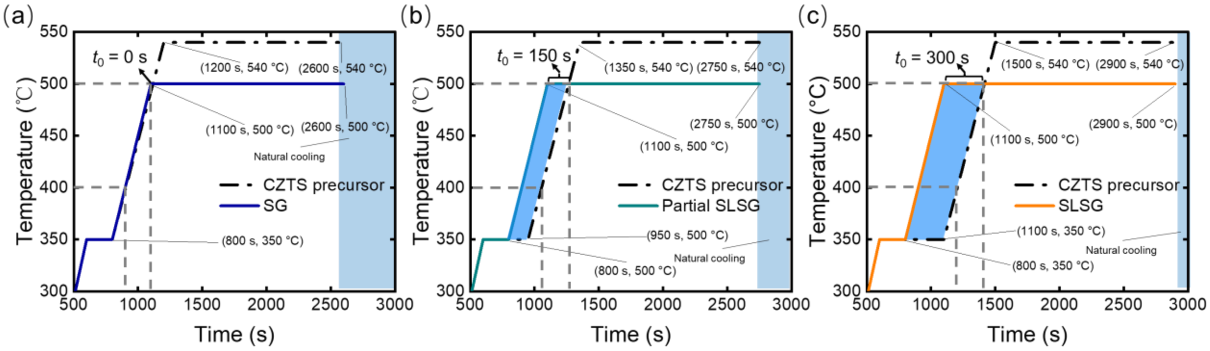

Supplementary Figure 3. Time-dependent temperature evolution of Se source (full line) and the precursor film (dash line): (a) SG, (b) Partial SLSG and (c) SLSG.

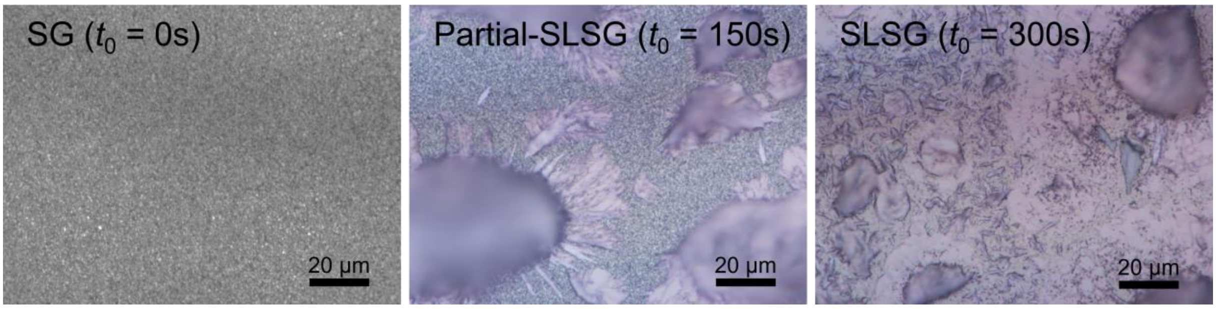

Supplementary Figure 4. Optical microscope images of SG, Partial SLSG, SLSG samples. When t_0 = 0 s, no liquid Se is observed on the sample surface; When t_0 = 150 s, the film is partially covered by Se; As t_0 reaches 300 s, the sample is entirely covered by Se.

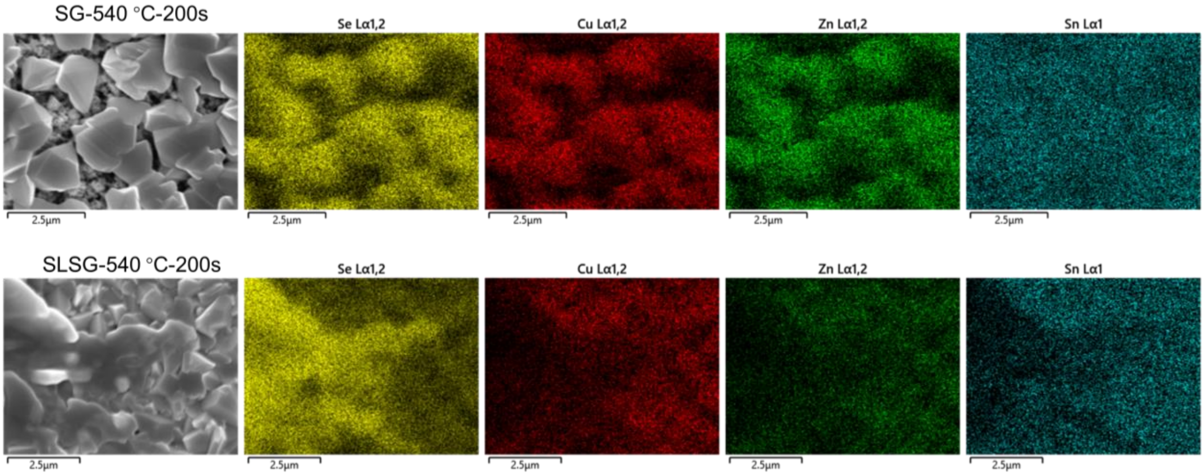

Supplementary Figure 5. SEM EDX characterizations of SG-540°C-200s and SLSG-540°C-200s samples. For the SLSG sample, an accumulation of Se elements can be clearly seen in the left region, which also shows a shapeless morphology, indicating the existence of elemental Se.

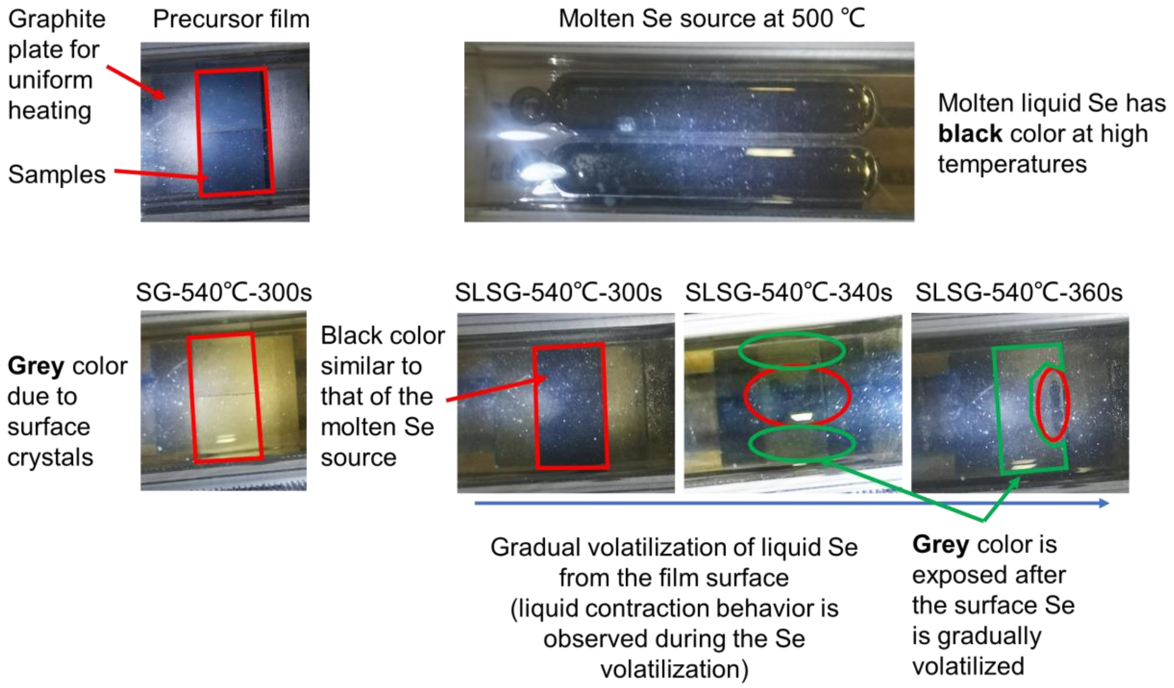

Supplementary Figure 6. Photographs of precursor films before selenization and the selenized samples (SG and SLSG) in-situ taken during the selenization process (540 °C for 300 s). The SG sample shows a grey color, a typical phenomenon for polycrystal film surface. For the SLSG sample, it shows a black color, which is similar to that of the molten Se source, indicating the liquid Se covered on the film. At longer time, the liquid Se gradually volatilized and the grey color of the film surface was exposed.

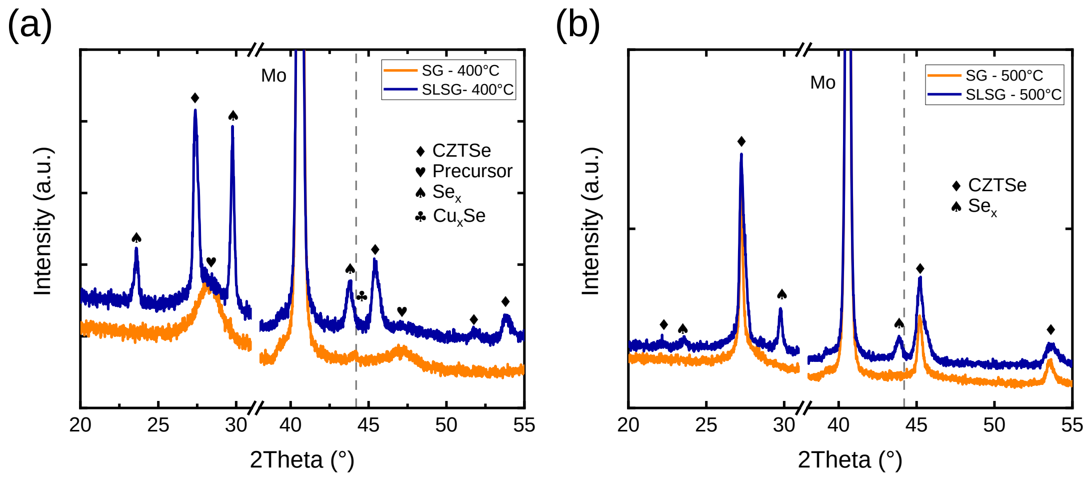

Supplementary Figure 7. XRD patterns of SG and SLSG samples in different intermediate stages: (a) 400 °C and (b) 500 °C. Three characteristics XRD peaks of Se_x can be clearly seen in the SLSG sample (~23.6°, 29.8°, 43.7°, referring to JADE PDF#06-0362). In the SG-400 °C sample, a small XRD peak corresponding to the $Cu_{2-x}Se$ phase can be seen at about 44.3° (JADE PDF#06-0680), which is consistent with the Raman result. In addition, the SLSG sample shows more obvious CZTSSe XRD peaks, indicating that the crystallization of CZTSSe has been facilitated.

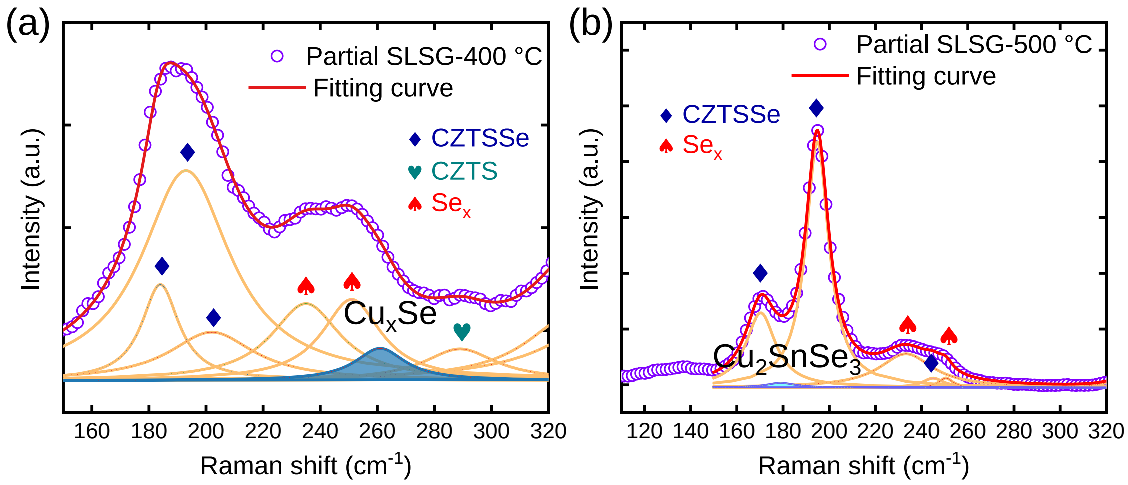

Supplementary Figure 8. Raman spectra of (a) Partial SLSG-400 °C and (b) Partial SLSG-500 °C. The circles show the raw data, and red lines give the fitting results. The signal of Cu_xSe still exists in Partial SLSG-400°C sample and a weak signal of Cu_2SnSe_3 can also be seen in the Partial SLSG-500°C sample.

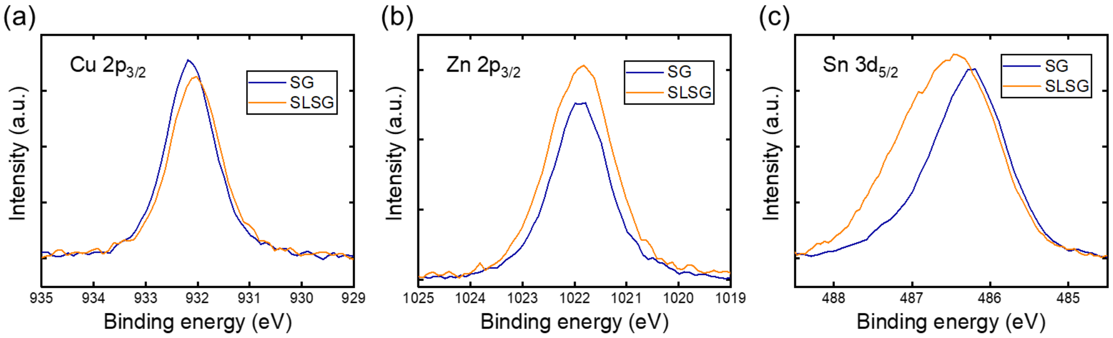

Supplementary Figure 9. XPS spectra of SG and SLSG: (a) Cu $2P_{3/2}$, (b) Zn $2P_{3/2}$, and (c) Sn $3d_{5/2}$.

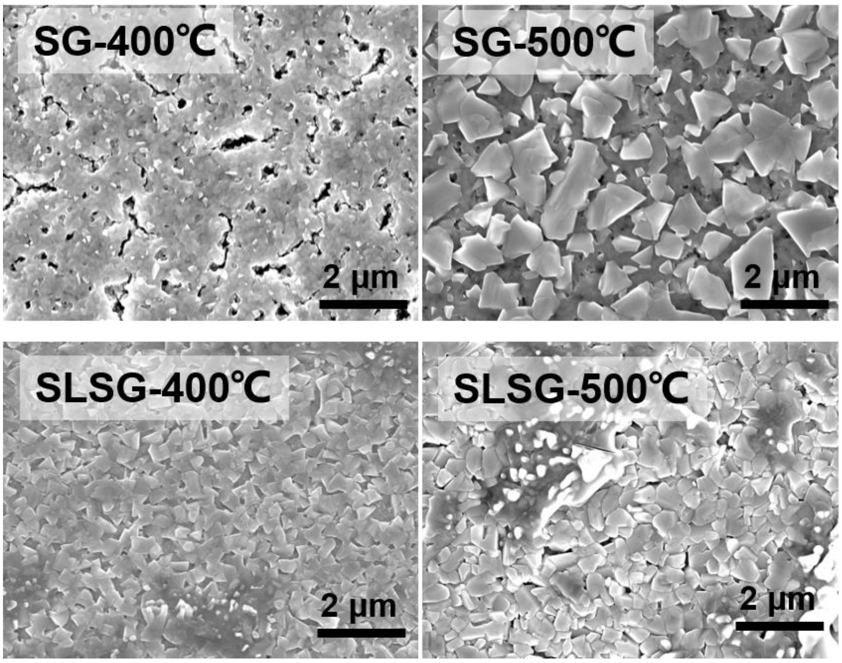

Supplementary Figure 10. The evolution of morphology of ACZTSSe films in SG and SLSG routes. The sample adopts the SLSG route owns more nucleation sites and a more compact morphology.

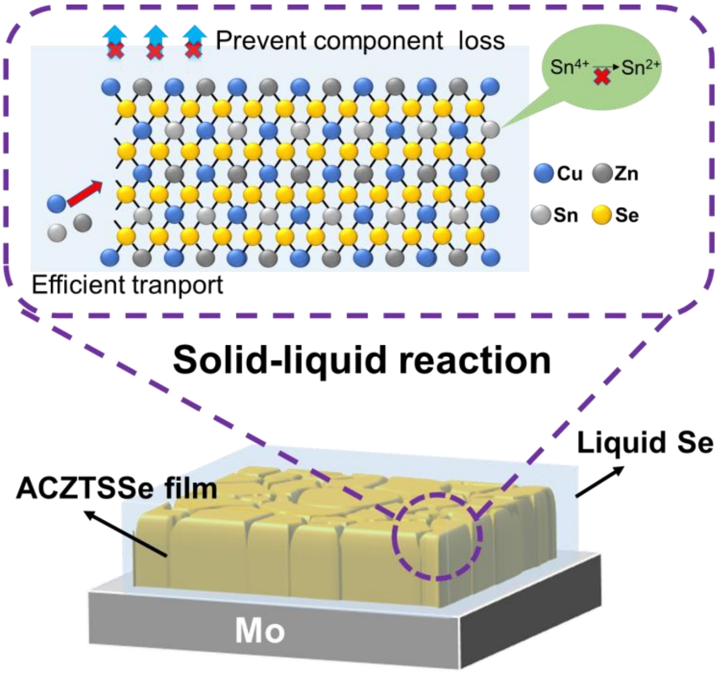

Supplementary Figure 11. The schematic of liquid Se assisted selenization of ACZTSSe. Sufficient Se could realize an efficient mass transport, suppress the variations of chemical state and compositions of the sample.

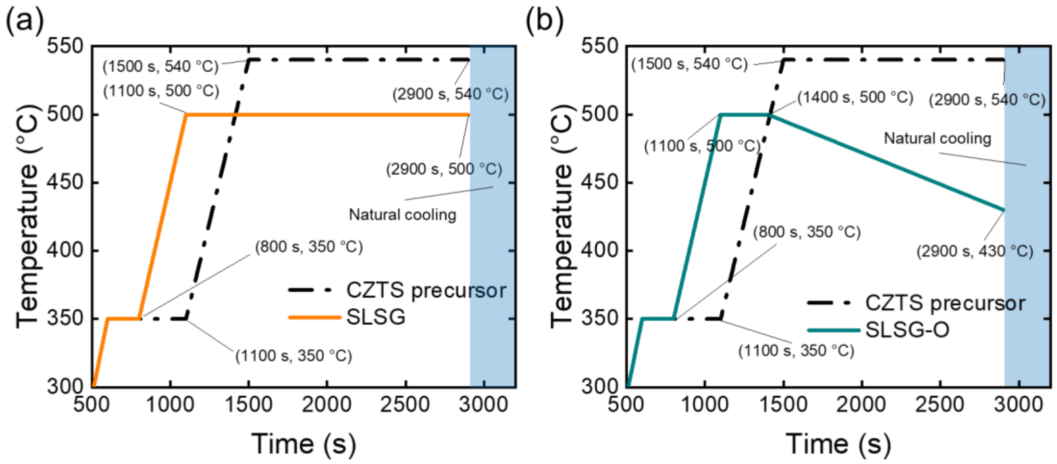

Supplementary Figure 12. Time-dependent temperature evolution designed in volatilization optimization experiments of SLSG selenization: (a) SLSG and (b) SLSG-O. The volatilization rate is regulated by varying temperature declining rate of Se source. The dash line represents temperature of the film sample, and the solid lines represent Se source temperature. SLSG has no decline in temperature of Se source; SLSG-O has the optimized declining rate.

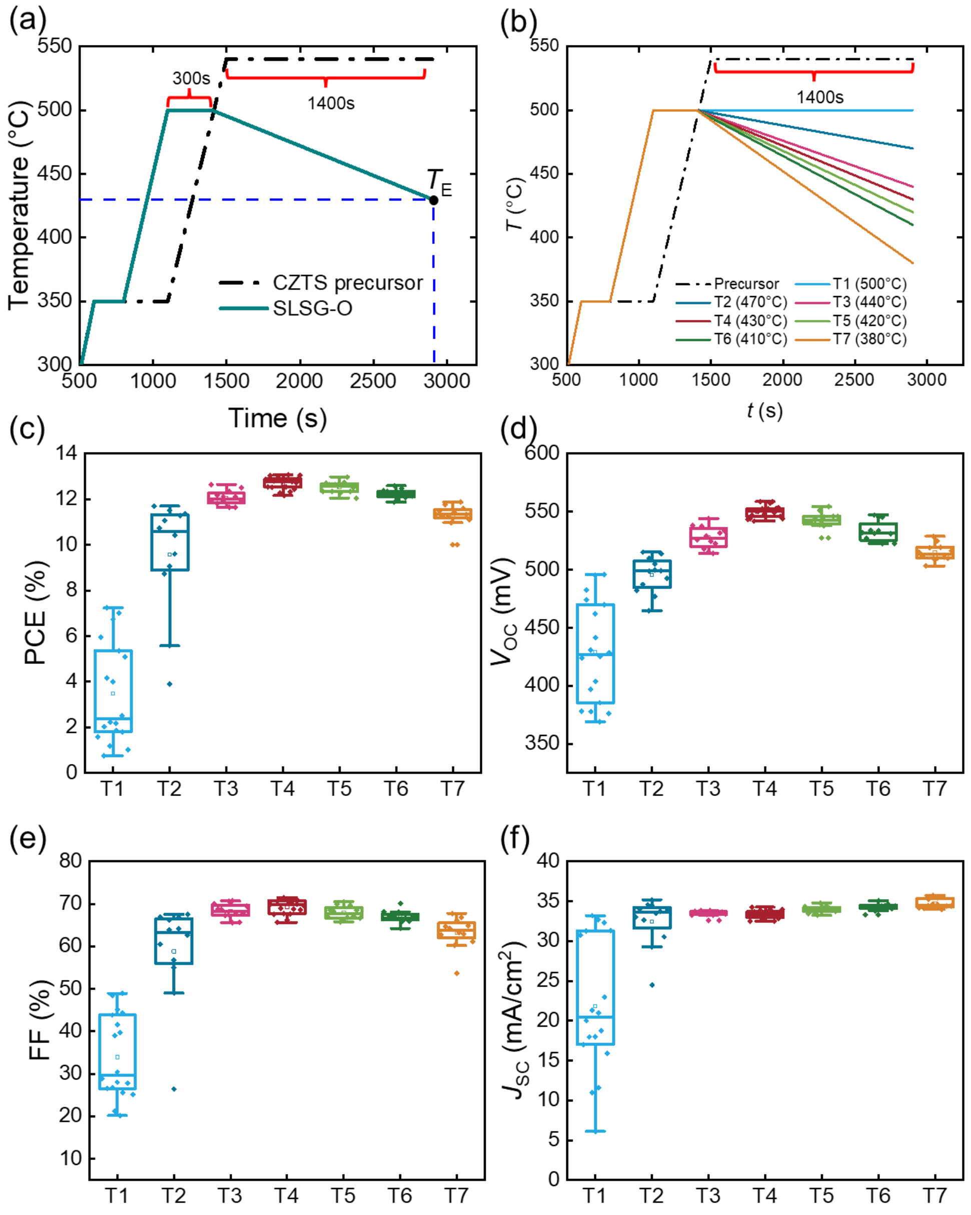

Supplementary Figure 13. (a) The schematic temperature profile of SLSG-O. T_E is the ending temperature of the Se source and is also the main variable in the optimization experiment of SLSG-O selenization. (b) The temperature profiles of SLSG-O with different T_E, which is labelled by T1-7. (c-f) Device performance of the cells under different T_E conditions. From T3 to T6, the cells all have average PCE of higher than 12%, indicating a wide experiment window.

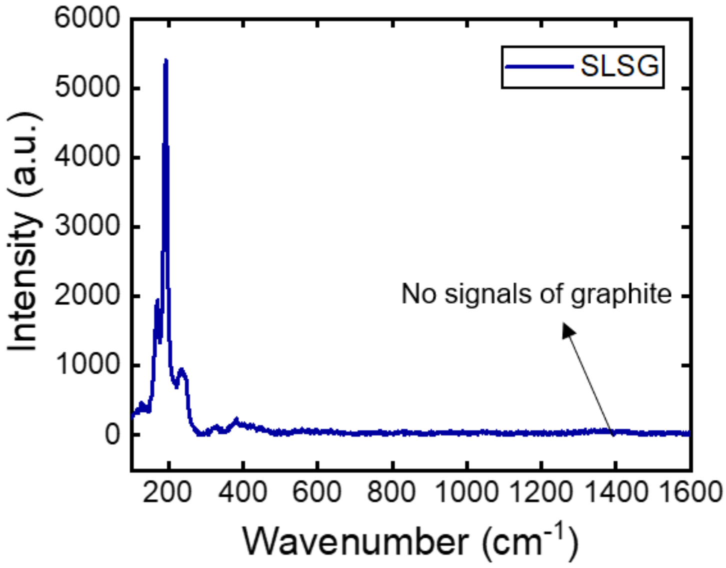

Supplementary Figure 14. Raman spectrum of the bottom amorphous layer in SLSG sample. And there are no signals around 1350 cm^{-1} and 1580 cm^{-1}, suggesting the absence of graphite-like carbon.

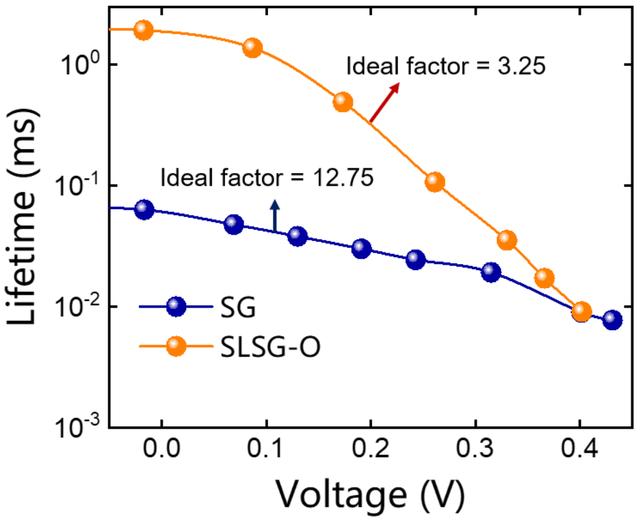

Supplementary Figure 15. Voltage-dependent photovoltage lifetimes of SG and SLSG-O samples.

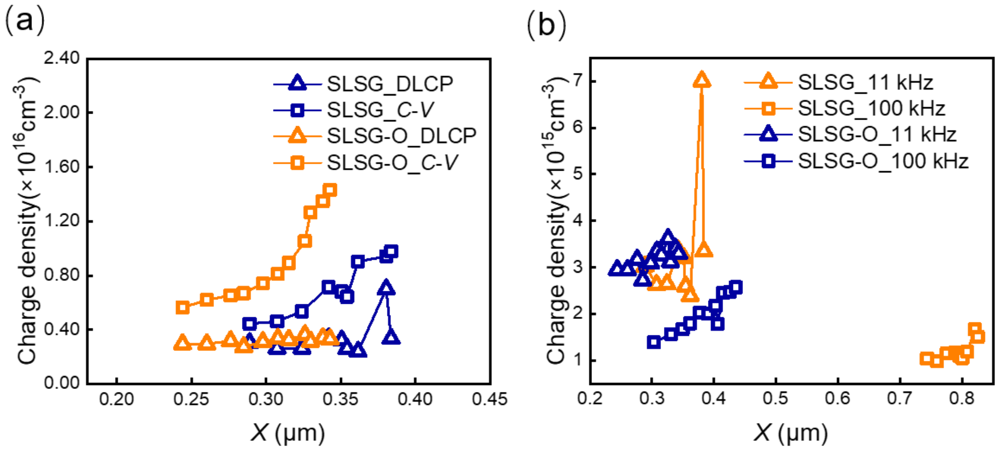

Supplementary Figure 16. (a) DLCP and *C*-*V* results of SLSG and SLSG-O samples at 11 kHz. (b) DLCP results of SLSG and SLSG-O at 11 kHz and 100 kHz. The depletion-region width of SLSG increases by about 0.44 μm with the frequency increasing to 11 KHz, indicating the existence of massive non-uniform bulk defects.

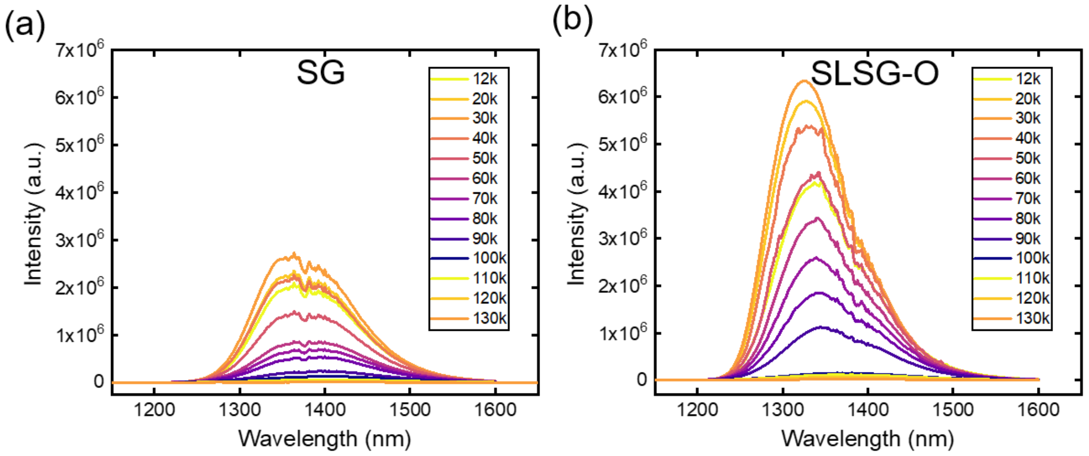

Supplementary Figure 17. Temperature-dependent PL spectra: (a) SG and (b) SLSG-O samples.

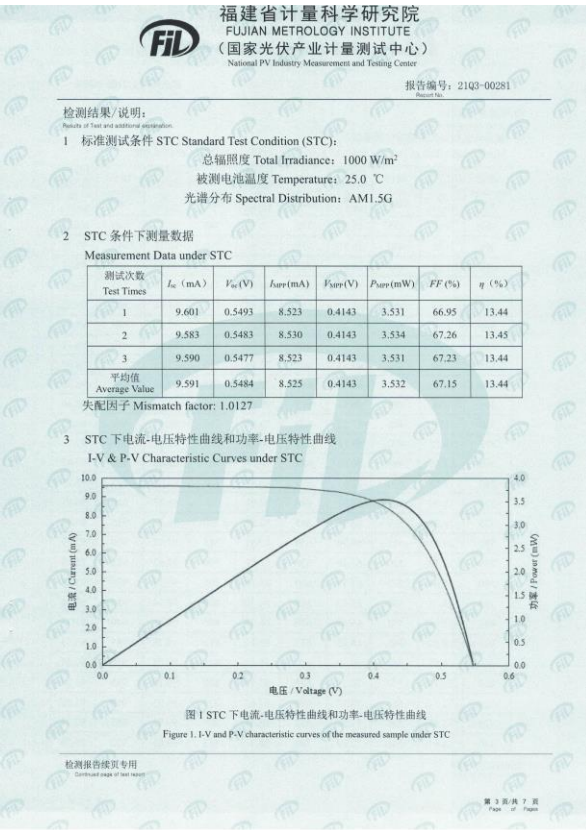

福建省计量科学研究院
FUJIAN METROLOGY INSTITUTE
（国家光伏产业计量测试中心）
National PV Industry Measurement and Testing Center

报告编号：21Q3-00281
Report No.

检测结果/说明：
Results of Test and additional explanation.

1 标准测试条件 STC Standard Test Condition (STC)：

总辐照度 Total Irradiance：1000 W/m^2
被测电池温度 Temperature：25.0 ℃
光谱分布 Spectral Distribution：AM1.5G

2 STC 条件下测量数据
Measurement Data under STC

测试次数 Test Times	I_{sc}（mA）	V_{oc}(V)	I_{MPP}(mA)	V_{MPP}(V)	P_{MPP}(mW)	FF (%)	η（%）
1	9.601	0.5493	8.523	0.4143	3.531	66.95	13.44
2	9.583	0.5483	8.530	0.4143	3.534	67.26	13.45
3	9.590	0.5477	8.523	0.4143	3.531	67.23	13.44
平均值 Average Value	9.591	0.5484	8.525	0.4143	3.532	67.15	13.44

失配因子 Mismatch factor: 1.0127

3 STC 下电流-电压特性曲线和功率-电压特性曲线
I-V & P-V Characteristic Curves under STC

图 1 STC 下电流-电压特性曲线和功率-电压特性曲线
Figure 1. I-V and P-V characteristic curves of the measured sample under STC

检测报告续页专用
Continued page of test report

第 3 页/共 7 页
Page of Pages

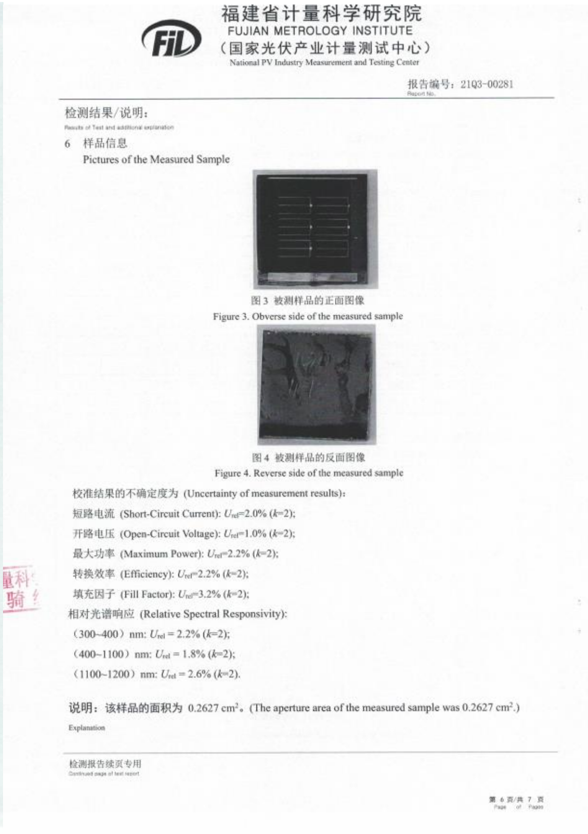

福建省计量科学研究院
FUJIAN METROLOGY INSTITUTE
（国家光伏产业计量测试中心）
National PV Industry Measurement and Testing Center

报告编号：21Q3-00281
Report No.

检测结果/说明：
Results of Test and additional explanation

6 样品信息
Pictures of the Measured Sample

图 3 被测样品的正面图像
Figure 3. Obverse side of the measured sample

图 4 被测样品的反面图像
Figure 4. Reverse side of the measured sample

校准结果的不确定度为 (Uncertainty of measurement results)：
短路电流 (Short-Circuit Current): U_{rel}=2.0% (k=2);
开路电压 (Open-Circuit Voltage): U_{rel}=1.0% (k=2);
最大功率 (Maximum Power): U_{rel}=2.2% (k=2);
转换效率 (Efficiency): U_{rel}=2.2% (k=2);
填充因子 (Fill Factor): U_{rel}=3.2% (k=2);
相对光谱响应 (Relative Spectral Responsivity):
（300~400）nm: U_{rel} = 2.2% (k=2);
（400~1100）nm: U_{rel} = 1.8% (k=2);
（1100~1200）nm: U_{rel} = 2.6% (k=2).

说明：该样品的面积为 0.2627 cm^2。(The aperture area of the measured sample was 0.2627 cm^2.)
Explanation

检测报告续页专用
Continued page of test report

第 6 页/共 7 页
Page of Pages

Supplementary Figure 18. Certification report of the champion ACZTSSe solar cell given by NPVM.

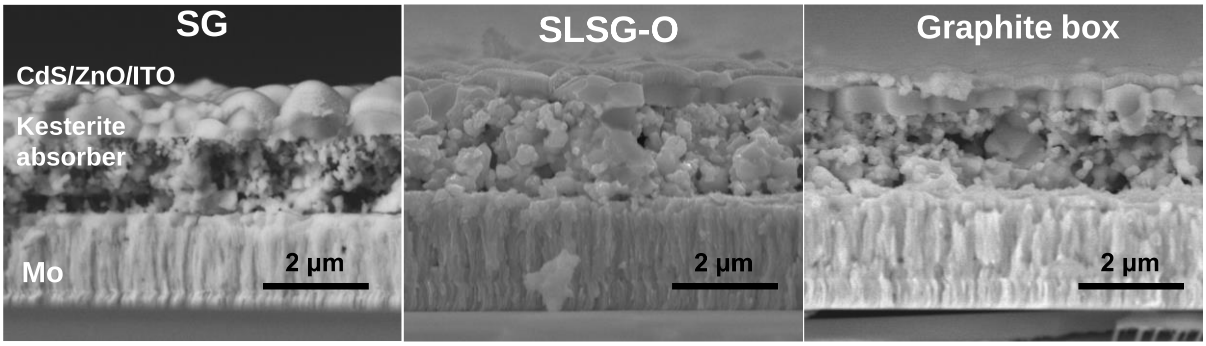

Supplementary Figure 19. Cross-sectional SEM images of the completed cells with the Kesterite absorber fabricated by different methods. It can be seen that the SLSG-O sample has the best cross-sectional morphology, especially the fine grain layer has much larger grain size.

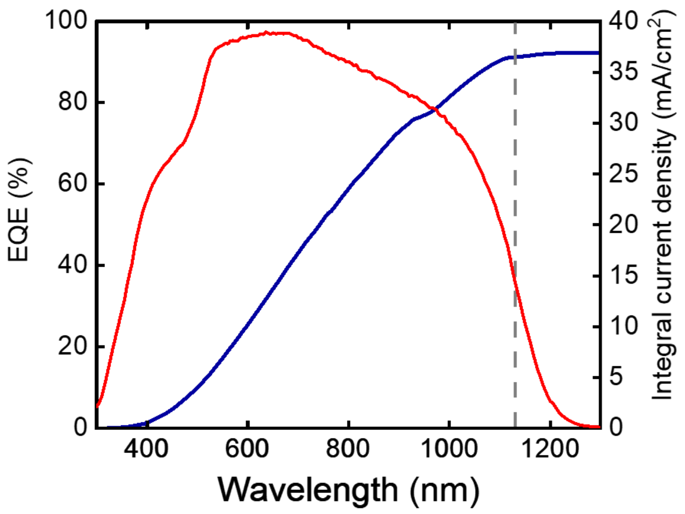

Supplementary Figure 20. External quantum efficiency (EQE) spectrum of the cell. The derived E_g is 1.10 eV (maximum position of -dEQE/dλ) and the integral current density is 37.0 mA/cm^2.

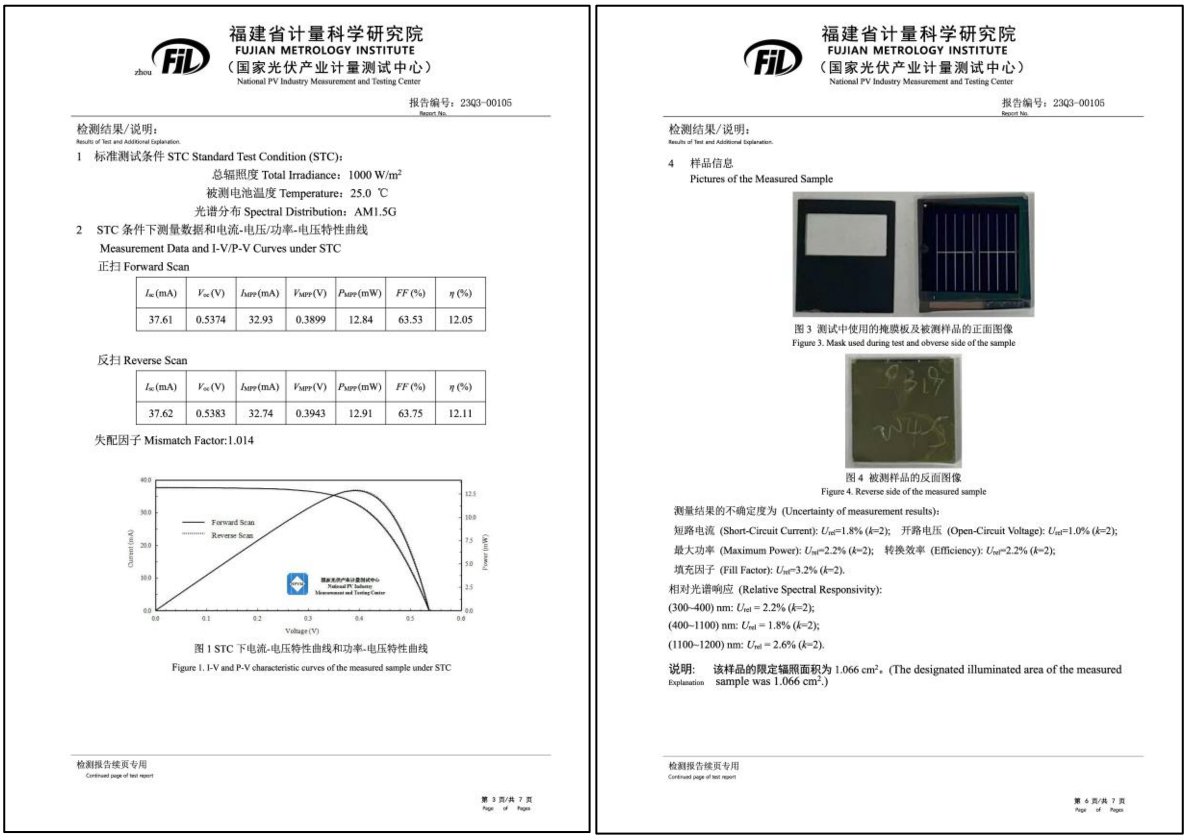

福建省计量科学研究院
FUJIAN METROLOGY INSTITUTE
（国家光伏产业计量测试中心）
National PV Industry Measurement and Testing Center

报告编号：23Q3-00105
Report No.

检测结果/说明：
Results of Test and Additional Explanation.

1 标准测试条件 STC Standard Test Condition (STC)：

总辐照度 Total Irradiance：1000 W/m²
被测电池温度 Temperature：25.0 ℃
光谱分布 Spectral Distribution：AM1.5G

2 STC 条件下测量数据和电流-电压/功率-电压特性曲线
Measurement Data and I-V/P-V Curves under STC

正扫 Forward Scan

I_{sc} (mA)	V_{oc} (V)	I_{MPP} (mA)	V_{MPP} (V)	P_{MPP} (mW)	FF (%)	η (%)
37.61	0.5374	32.93	0.3899	12.84	63.53	12.05

反扫 Reverse Scan

I_{sc} (mA)	V_{oc} (V)	I_{MPP} (mA)	V_{MPP} (V)	P_{MPP} (mW)	FF (%)	η (%)
37.62	0.5383	32.74	0.3943	12.91	63.75	12.11

失配因子 Mismatch Factor:1.014

图 1 STC 下电流-电压特性曲线和功率-电压特性曲线
Figure 1. I-V and P-V characteristic curves of the measured sample under STC

检测报告续页专用
Continued page of test report

第 3 页/共 7 页

福建省计量科学研究院
FUJIAN METROLOGY INSTITUTE
（国家光伏产业计量测试中心）
National PV Industry Measurement and Testing Center

报告编号：23Q3-00105
Report No.

检测结果/说明：
Results of Test and Additional Explanation.

4 样品信息
Pictures of the Measured Sample

图 3 测试中使用的掩膜板及被测样品的正面图像
Figure 3. Mask used during test and obverse side of the sample

图 4 被测样品的反面图像
Figure 4. Reverse side of the measured sample

测量结果的不确定度为 (Uncertainty of measurement results)：
短路电流 (Short-Circuit Current): U_{rel}=1.8% (k=2); 开路电压 (Open-Circuit Voltage): U_{rel}=1.0% (k=2);
最大功率 (Maximum Power): U_{rel}=2.2% (k=2); 转换效率 (Efficiency): U_{rel}=2.2% (k=2);
填充因子 (Fill Factor): U_{rel}=3.2% (k=2).
相对光谱响应 (Relative Spectral Responsivity):
(300~400) nm: U_{rel} = 2.2% (k=2);
(400~1100) nm: U_{rel} = 1.8% (k=2);
(1100~1200) nm: U_{rel} = 2.6% (k=2).

说明: **该样品的限定辐照面积为** 1.066 cm²。(The designated illuminated area of the measured sample was 1.066 cm².)
Explanation

检测报告续页专用
Continued page of test report

第 6 页/共 7 页

Supplementary Figure 21. Certification report of the large-area solar cell given by NPVM.

Supplementary Table 1. Detailed parameters used in the simulation.

Symbol & formula	Value	Description
ΔH	95.48 [kJ/mol]	latent heat of vaporization[2]
A	6.33714	constant for Antoine equation[1,6]
B	6588.125	constant for Antoine equation[1,6]
C	86.633	constant for Antoine equation[1,6]
Rs	0.32* 461.5 [J/kg/K]	specific gas constant
M_{Se}	157.92e-3 [kg/mol]	molecular weight of Se_2
sigma	70 [mN/m]	surface tension
ρ	4.81 [g/cm^3]	Se density
T_{ext}	(273.15+550) [K]	environment temperature
hh	5 [W/(m^2·K)]	convective heat transfer coefficient
V_A	31.1	molal volumes at the normal boiling point (N_2) [7]
V_B	60	molal volumes at the normal boiling point (Se_2)[7]
M_A	28	ordinary mol. Weight of N_2[7]
M_B	158	ordinary mol. Weight of Se_2[7]
P	0.1 [MPa]	partial pressure of N_2
Q	50 [sccm]	flux of carrier gas
L_1	3.5 [cm]	radius of graphite box
H_1	0.7 [cm]	height of graphite box
r	0.15 [cm]	radius of Se droplet
l_1	2 [cm]	width of precursor
h1	0.2 [cm]	height of precursor
d_1	1.8 [cm]	distance between Se source and precursor

L_2	40 [cm]	length of rectangle quartz tube
H_2	1.8 [cm]	height of rectangle quartz tube
l_2	10 [cm]	length of Se source
h_2	1 [cm]	height of Se source
l_3	2 [cm]	length of precursor
h_3	0.2 [cm]	height of precursor
d_2	21 [cm]	distance between Se source and precursor

Supplementary Table 2. The detailed performance data of SG, SLSG, and SLSG-O cells.

SG				SLSG				SLSG-O			
J_{SC} (mA/cm^2)	V_{OC} (mV)	FF	PCE (%)	J_{SC} (mA/cm^2)	V_{OC} (mV)	FF	PCE (%)	J_{SC} (mA/cm^2)	V_{OC} (mV)	FF	PCE (%)
34.1	499.9	0.58	9.8	21.0	369.1	0.28	2.2	33.3	552.0	0.66	12.2
33.8	503.2	0.59	10.1	18.0	385.5	0.27	1.9	32.9	547.9	0.71	12.8
33.9	510.9	0.61	10.6	11.6	396.9	0.26	1.2	33.5	552.5	0.68	12.5
33.0	504.9	0.60	9.9	18.0	377.8	0.27	1.8	32.8	552.7	0.68	12.3
33.9	506.8	0.60	10.3	21.3	376.2	0.28	2.2	34.0	557.6	0.69	13.1
33.6	500.2	0.57	9.6	22.9	378.3	0.29	2.5	33.9	558.8	0.69	13.0
32.4	493.8	0.60	9.6	32.7	495.1	0.42	6.7	33.6	558.6	0.66	12.3
34.2	520.6	0.54	9.5	33.2	482.5	0.45	7.2	33.3	550.1	0.70	12.8
33.4	505.6	0.59	10.0	32.3	495.9	0.44	7.0	32.5	549.2	0.72	12.8
34.0	496.1	0.62	10.5	6.1	403.8	0.30	0.8	33.1	550.6	0.70	12.8
33.5	493.4	0.60	9.9	18.7	441.6	0.48	4.0	33.4	552.8	0.68	12.4
33.9	505.0	0.59	10.1	20.0	425.3	0.49	4.2	33.7	541.9	0.69	12.5
33.2	493.4	0.59	9.6	31.3	430.8	0.40	5.4	32.8	553.6	0.70	12.7
33.9	492.9	0.63	10.6	31.3	428.6	0.44	6.0	34.2	546.8	0.70	13.0
34.3	505.0	0.57	9.8	30.8	424.1	0.39	5.1	33.2	545.6	0.71	12.9
34.7	503.0	0.54	9.5	11.0	462.0	0.20	1.0	32.5	544.0	0.71	12.6
33.8	498.7	0.59	9.9	17.0	474.0	0.25	2.0	34.0	544.0	0.71	13.1
33.2	501.9	0.61	10.1	15.9	470.0	0.21	1.6	33.4	543.3	0.71	12.9

Supplementary References

1 Thomson, G. W. THE ANTOINE EQUATION FOR VAPOR-PRESSURE DATA. *Chemical reviews* **38**, 1-39 (1949).

2 Yamdagni, R. & Porter, R. F. Mass Spectrometric and Torsion Effusion Studies of the Evaporation of Liquid Selenium. *Journal of The Electrochemical Society* **115**, 601 (1968).

3 Lagerstrom, P. A. *Laminar flow theory*. (Princeton University Press, 1996).

4 Poirier, D. R., Geiger, G. H. & Poirier, D. R. Fick's Law and diffusivity of materials. *Transport Phenomena in Materials Processing*, 419-461 (2016).

5 Incropera, F. P., DeWitt, D. P. & Bergman, T. L. *Fundamentals of heat and mass transfer*. Vol. 6 (Wiley, 1996.).

6 *NIST Chemistry WebBook*, <https://webbook.nist.gov/cgi/cbook.cgi?ID=C7782492&Units=SI&Mask=4#Thermo-Phase> (2023).

7 Gilliland, E. R. Diffusion Coefficients in Gaseous Systems. *Industrial and engineering chemistry* **26**, 6 (1934).
