# Supplementary material for: Controlling Selenization Equilibrium Enables High-Quality Kesterite Absorbers for Efficient Solar Cells
Source: Nat Commun. 2023 Oct 20;14:6650. doi: 10.1038/s41467-023-42460-7 (PMC10589234; doi:10.1038/s41467-023-42460-7)
Supplement: Supplementary file 3 — Reporting Summary [file 41467_2023_42460_MOESM3_ESM.pdf]

nature research

Corresponding author(s): Qingbo Meng, Yanhong Luo, Jiangjian Shi

Solar Cells Reporting Summary

Nature Research wishes to improve the reproducibility of the work that we publish. This form is intended for publication with all accepted papers reporting the characterization of photovoltaic devices and provides structure for consistency and transparency in reporting. Some list items might not apply to an individual manuscript, but all fields must be completed for clarity.

For further information on Nature Research policies, including our data availability policy, see Authors & Referees.

Experimental design

Please check: are the following details reported in the manuscript?

1. Dimensions

Item	Yes	No	Comment
Area of the tested solar cells	☒	☐	Given in Experimental section and in the certification report.
Method used to determine the device area	☒	☐	Given in Experimental section and in the certification report.

2. Current-voltage characterization

Item	Yes	No	Comment
Current density-voltage (J-V) plots in both forward and backward direction	☐	☒	Kesterite solar cells do not have hysteresis behavior. One-direction scanning is enough to give reliable result.
Voltage scan conditions<br>*For instance: scan direction, speed, dwell times*	☒	☐	Given in Experimental section.
Test environment<br>*For instance: characterization temperature, in air or in glove box*	☒	☐	Given in Experimental section.
Protocol for preconditioning of the device before its characterization	☒	☐	Given in the experimental section.
Stability of the J-V characteristic<br>*Verified with time evolution of the maximum power point or with the photocurrent at maximum power point; see ref. 7 for details.*	☐	☒	Kesterite solar cells do not have hysteresis. And in the certification process, maximum power output had been traced for several minutes to confirm the cell efficiency.

3. Hysteresis or any other unusual behaviour

Item	Yes	No	Comment
Description of the unusual behaviour observed during the characterization	☐	☒	Kesterite solar cells do not have hysteresis or other unusual behaviour.
Related experimental data	☐	☒	Kesterite solar cells do not have hysteresis or other unusual behaviour.

4. Efficiency

Item	Yes	No	Comment
External quantum efficiency (EQE) or incident photons to current efficiency (IPCE)	☒	☐	Given in Supplementary Materials.
A comparison between the integrated response under the standard reference spectrum and the response measure under the simulator	☒	☐	Given in Manuscripit and Supplementary Materials.
For tandem solar cells, the bias illumination and bias voltage used for each subcell	☐	☒	No tandem solar cell was studied.

5. Calibration

Item	Yes	No	Comment
Light source and reference cell or sensor used for the characterization	☒	☐	Given in Experimental Section.
Confirmation that the reference cell was calibrated and certified	☒	☐	Given in Experimental Section.

Calculation of spectral mismatch between the reference cell and the devices under test

- [x] Yes
- [ ] No

Given in the certification report.

6. Mask/aperture

Size of the mask/aperture used during testing

- [x] Yes
- [ ] No

Given in certification report.

Variation of the measured short-circuit current density with the mask/aperture area

- [ ] Yes
- [x] No

No such phenomenon in Kesterite solar cells.

7. Performance certification

Identity of the independent certification laboratory that confirmed the photovoltaic performance

- [x] Yes
- [ ] No

Certified in NPVM.

A copy of any certificate(s)
Provide in Supplementary Information

- [x] Yes
- [ ] No

Given in Supplementary Materials.

8. Statistics

Number of solar cells tested

- [x] Yes
- [ ] No

18 cells are used for statistic analysis.

Statistical analysis of the device performance

- [x] Yes
- [ ] No

Given in Figure 3(g).

9. Long-term stability analysis

Type of analysis, bias conditions and environmental conditions
For instance: illumination type, temperature, atmosphere humidity, encapsulation method, preconditioning temperature

- [ ] Yes
- [x] No

No stability problem has been reported in Kesterite solar cells.
